# Comparison of physical activity levels and dietary habits between women with polycystic ovarian syndrome and healthy controls of reproductive age: a case-control study

**DOI:** 10.1186/s12905-023-02866-3

**Published:** 2024-01-08

**Authors:** Muhammad Salman Butt, Javeria Saleem, Rubeena Zakar, Sobia Aiman, Gul Mehar Javaid Bukhari, Florian Fischer

**Affiliations:** 1 Department of Public Health, University of the Punjab, Lahore, Pakistan; 2 Akhtar Saeed Medical and Dental College, Lahore, Pakistan; 3 Department of Community Medicine, Federal Medical and Dental College Islamabad, Islamabad, Pakistan; 4 Institute of Public Health, Charité – Universitätsmedizin Berlin, Charitéplatz 1, Berlin, 10117 Germany

**Keywords:** Polycystic ovarian syndrome, Physical activity, Dietary habits, Glycaemic index, Glycaemic load

## Abstract

**Background:**

Polycystic ovarian syndrome (PCOS) is a reproductive hormonal anomaly prevalent among women of reproductive age, with an alarmingly high prevalence of 52% among Pakistani women. This study aims to compare the daily physical activity and dietary habits of women with PCOS with age-matched healthy controls living in Lahore, Pakistan.

**Methods:**

A case–control study design was used to collect data from a private hospital situated in Lahore, Pakistan. Data was collected from 115 participants of reproductive age (18–45 years) using a researcher-administered questionnaire. Demographic variables, reproductive characteristics, anthropometric measurements, and seven days of physical activity levels using the international physical activity questionnaire (IPAQ-Short version) and seven days of dietary intake using the food frequency questionnaire (7 days-FFQ) were used to measure the dietary habits of the participants. Mosby’s Nutritac v4.0 software was used to estimate the macronutrients, vitamins, and minerals present in dietary intake. The glycaemic index and glycaemic load were calculated to compare the quality and quantity of carbohydrate consumption between the two groups.

**Results:**

The 49 PCOS cases, newly identified using the Rotterdam criteria, mean age 24.63 years (SD ± 4.76), and 66 healthy controls, mean age 23.24 years (SD ± 5.45), were compared. A significant difference (*p* ≤ 0.05) was found for reproductive characteristics, daily physical activity, and polyunsaturated fat and vitamin intake between the two groups. A binary logistic regression analysis showed that food with a low glycaemic index (GI ≤ 40) reduced the odds of PCOS occurrence by OR = 1.94. Similarly, food nutrients with a low glycaemic load (GL ≤ 10) can reduce PCOS occurrence by OR = 1.60.

**Conclusion:**

The daily physical activity levels and dietary habits of women of reproductive age can influence their reproductive characteristics and polycystic ovarian morphology. A diet with a low glycaemic load and index can produce beneficial reproductive health effects among women of reproductive age.

**Supplementary Information:**

The online version contains supplementary material available at 10.1186/s12905-023-02866-3.

## Background

Polycystic ovarian syndrome (PCOS) is a reproductive hormonal anomaly prevalent among women of reproductive age. A global prevalence of PCOS of 6–8% is estimated when diagnosed using the National Institute of Health criteria [1]. PCOS can be considered a multifactorial endocrinopathy disease that can further develop into hyperinsulinemia, reduced insulin sensitivity, and metabolic syndrome [2]. The worldwide prevalence of PCOS differs among various ethnic groups and declines with increasing age. A prevalence of 33% in British [3], 21–23% in Australian and New Zealand [4], and 21.6% in Finnish [5] women of reproductive age has been found. An alarmingly high prevalence of 52% has been reported among Pakistani women [6].

The Rotterdam criteria 2003 are considered one of the globally accepted sets of criteria for PCOS diagnosis, and the presence of any two clinical features out of the following three is essential for PCOS diagnoses [7]: (i) oligomenorrhea or anovulatory menstrual pattern (cycle length ≥ 35 days), (ii) biochemical or clinical signs of hyperandrogenism, and (iii) polycystic ovarian morphology. Concurrent occurrence of regular menstrual cycles along with ovulatory dysfunction has also been observed among women with PCOS [2]. Clinical signs of hyperandrogenism, including hirsutism, androgen alopecia, acne, and virilization leading to insulin resistance, are also common [1].

A transvaginal ultrasonogram (TVS) examination shows polycystic ovarian morphology (PCOM) with reraised ovarian volume (≥ 10 mL) and 12 follicles (2–9 mm) among most women with PCOS [7, 8]. The sole presence of PCOM occasionally becomes confused with PCOS, whereas 30% of PCOM cases report normal reproductive function and menstruation [9]. It has been recommended not to label PCOS merely on the basis of PCOM unless a concurrent presence of hyperandrogenism and menstrual irregularities is confirmed, in line with the Rotterdam criteria [10, 11]. Oligo-/ anovulation is the most common ovulatory dysfunction among women with PCOS. Some women also manifest ovulatory dysfunction with normal menstrual cycles [7].

Women with PCOS are also found to have insulin resistance (IR) and hyperinsulinemia, which can further lead to metabolic syndrome and other comorbidities [12, 13]. The synergetic actions of metabolic syndrome and PCOS affect hepatic functions and glucose production to cause insulin resistance and diabetes mellitus type II [14]. Previous studies have documented beneficial effects on reproductive functions among women with PCOS from improving their daily physical activity levels. Aerobic training for three months was found to reduce menstrual irregularities among 70% of women with PCOS [15].

The international PCOS guidelines suggest moderate physical activity for 150 min per week and vigorous activity for 75 min per week for normal-weight women with PCOS. It has been suggested that obese women with PCOS should engage in moderate activity for 250 min per week and vigorous activity for 150 min per week, or an equivalent combination of both to obtain reproductive health benefits [10]. Regular physical activity has also been found to improve glycaemic control, metabolic functions, and quality of life among women with PCOS [16, 17].

Maximum oxygen consumption (VO_2_ max) is a commonly used experimental procedure to estimate physical fitness levels [18]. Several observational tools, including the International Physical Activity Questionnaire (IPAQ), have been established, primarily to estimate the physical activity levels and energy expenditure of an individual. The IPAQ short version is a globally accepted tool that considers both leisure and physical activity assessments to estimate energy expenditure by analysing the frequency, duration, and intensity of activities performed over the previous seven days [19].

Obesity and weight gain are indispensable factors implicated in PCOS aetiology. A 30% prevalence has been reported for obesity and insulin resistance among women with PCOS [20]. The beneficial effects of a healthy diet on PCOS development are fascinating to contemplate and are considered the first line of treatment for PCOS [21]. However, the effects of dietary intake and macronutrient food composition have not been introduced in any international guidelines and have yet to make their reputation in PCOS management [10]. The exact dietary composition needed to facilitate PCOS remains ambiguous, as some studies comparing a normal protein diet with high protein intake [22], and a high-protein with a high-carbohydrate diet [23] showed non-significant PCOS-specific health outcomes.

Previous studies have focused on comparing sedentary lifestyles with common noncommunicable chronic diseases, such as cardiovascular, gastrointestinal, and psychological diseases. The alarming prevalence of PCOS among women of reproductive age has prompted researchers to compare the lifestyle patterns of women with PCOS with healthy controls. This study aims to compare physical activity and dietary patterns, including macronutrients, vitamins, minerals, glycaemic load (GL), and glycaemic index (GI), between women of reproductive age with PCOS and age-matched healthy controls living in Lahore, Pakistan.

## Methods

### Study design and setting

A case–control study design was used to collect data from a private outpatient health centre in Lahore, Pakistan. This study was extracted from a doctoral thesis project entitled: “Association of physical activity and dietary habits with vitamin D and anti-Müllerian hormone among polycystic ovarian syndrome women in Lahore, Pakistan” by the first author (MSB). A comparison of physical activity levels and dietary intake between women of reproductive age with PCOS and age-matched healthy controls is one of the prime objectives of this doctoral thesis. The data was collected between June 2019 and October 2021, and a consultant gynaecologist (SA) examined the cases and controls for possible recruitment to this study. The cases were recruited from the fertility/gynaecology outdoor facility, whereas the controls were selected from outpatient who were accompanying patients at surgical, medical, and paediatrics outpatient departments in the same private hospital.

### Study population

The women forming both the cases and controls were of reproductive age (15–45 years) and living in Lahore. Women with two out of three Rotterdam criteria, with both ovaries intact, and newly diagnosed with PCOS by the consultant gynaecologist were considered cases. A transvaginal pelvic ultrasonogram (TVS) was used (Toshiba Xario Prime, Crawley, UK) to confirm PCOM and ovary volume. The diagnostic criteria for PCOM were an ovarian volume > 10 cm^3^ or where the periphery of the central stroma had an appearance of ≥ 12 follicles [24]. The menstrual irregularities of women with PCOS were categorized into oligomenorrhea (≤ 8 periods/year, cycle duration ≥ 45 days), amenorrhea (no periods for longer than six months), and normal menstrual cycle (cycle duration 22–28 days). The modified Ferriman-Gallwey score (mFG ≥ 8) was used to identify clinical hyperandrogenism using hirsutism as a primary indicator [25, 26].

Age-matched controls were selected for the study from women visiting the same private hospital for treatment of their children or relatives. The controls had normal menstrual functions and no known chronic medical conditions. All the participants were healthy and not on any medications that could affect their reproductive hormones or body metabolism. Participants enrolled in any special diet or exercise or weight loss programme at the time or during the three months before the start of this study were also excluded.

### Sampling technique

The data was collected from 49 cases and 66 controls using a non-probability purposive sampling technique. The researchers tried to maintain a 1:2 case-to-control ratio by matching for age. When compared to a 1:1 ratio, a 1:2 ratio delivers greater statistical power. World Health Organization software v.2.0 was used for sample size calculation by estimating an odds ratio (OR) of 2 with a specified relative precision (ε) of 0.40. A PCOS prevalence of 50% was considered in Pakistan to anticipate an exposure probability to a specified disease (P1) of 0.50 [6]. An anticipated exposure probability to no disease (P2) was assumed to be 0.33 and was estimated at a confidence level (1-α) of 95%, as shown in Eq. 1.

1 $$n=\frac{{z}^{2}1-\alpha /2}{{\left[{log}_{e}\left(1-\epsilon \right)\right]}^{2}}\left[\frac{1}{P1 (1-P1)}+\frac{1}{P2(1-P2)}\right]$$

**Fig. 1:**
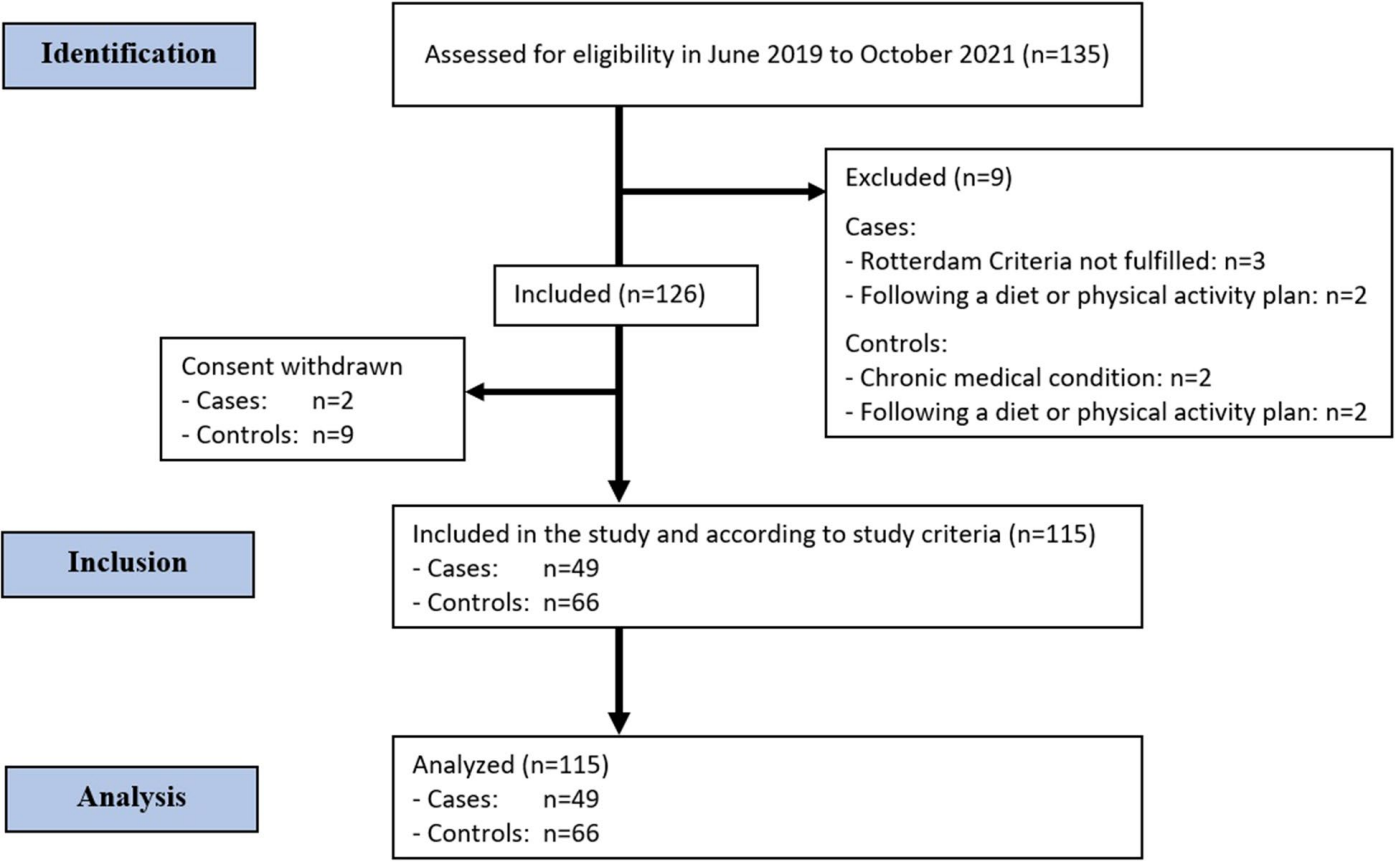
STROBE flow chart for participant selection and analysis

These selected parameters estimated a sample size of 126 for this case–control study. Consent was subsequently withdrawn by two cases and nine controls; thus, data was collected from 115 participants, as shown in Fig. 1.

### Data collection

The participants were briefed on the study’s objectives and asked for their consent to participate. The consultant gynaecologists and physicians clinically assessed cases and controls to record their menstrual cycles, clinical signs of hyperandrogenism, and medical history using a researcher-administered questionnaire. Cases were further investigated using TVS to confirm the PCOM. Baseline demographic and reproductive characteristics were collected using a self-administered questionnaire.

The lady health worker (LHW) used an outstretched tape measure placed at umbilical sites without additional pressure on the body surface to measure waist circumference (WC). The waist-to-hip ratio (W/H) was calculated after measuring the hip circumference at the broadest part. A digital weighing scale (Beurer BF 600) was used to measure weight in normal clothing, and the value was rounded to the nearest 100 g. A stadiometer (SECA 213) was used to measure height by placing it against a flat surface. The participants were asked to place their feet flat, keeping their legs straight and together at the centre of the back base of the board. The measurement was recorded to the nearest 0.5 cm by adjusting the headpiece over the head of the participant.

The short version of the International Physical Activity Questionnaire (IPAQ) was used to measure the physical activity and energy expenditure (MET-min/week) for the cases and controls. Physical activity and energy expenditure were classified into walking (low), moderate, and vigorous physical activities. The IPAQ has an interclass correlation coefficient reliability of 0.76–0.97 and a validity of 0.45–0.69 [19]. A structured food frequency questionnaire (FFQ) measuring a seven-day dietary pattern was developed with the help of a consultant nutritionist (see Supplementary material). The FFQ was designed for regional settings and contained 11 continental food categories and 33 food items frequently used in Lahore, Pakistan. The participants were briefed on the calculation of serving size or food consumed in grams. A pilot test with 25 participants was performed to measure the interclass correlation coefficient relation. A value of 0.697 was found using Cronbach’s alpha statistical testing.

Hyperandrogenism was measured using clinical signs by using the modified Ferriman-Gallwey (mFG) score by evaluating hirsutism. Nine androgen-sensitive body parts (upper thigh, lower and upper abdomen, lower and upper back, upper arm, chest, chin, and upper lip) were scored by the participants on a Likert scale (0–4) for hirsutism using a visual scoring tool. The recorded score for hyperandrogenism was categorized into severe (mFG score ≥ 25), moderate (mFG score 16–25), mild (mFG score 8–15), and normal (mFG score ≤ 8).

### Data analysis

The data was entered into an Excel spreadsheet on a daily basis for possible cleaning, verification, and correction after consultation with the gynaecologist and LHW. The IBM SPSS/PC v23.0 software package (SPSS, Inc., Chicago, IL, USA) was used to analyse the data. The case–control demographic, anthropometric, and reproductive characteristics data were categorized for descriptive analysis (mean [M] ± standard deviation [SD]) and frequency tabulation. The IPAQ-AUTOMATIC REPORT-English advanced version-self-admin short-Di Blasio & Izzicupo et al. Excel spreadsheet download from www.ipaq.ki.se was used to calculate the physical activity and energy expenditure. A cross-tabulation frequency table and M ± SD were created for comparisons between the two groups.

Mosby’s Nutritac v4.0 software was used to estimate the macronutrient, vitamin, and mineral values for the measured food items using the relevant food labels in the software, which were calculated for M ± SD comparisons between cases and controls. The nutrient data was entered into Excel software, and average nutrient consumption was measured to calculate the glycaemic load using Eq. 2.

2 $$Meal \,Glycaemic \,Index \,(GI)=\{[GIFoodA \,X \,g \,available \,carbohydrate \,(available \,CHO)Food \,A \,+ \,GIFood \,B \,X \,g \,available \,carbohydrate \,(available \,CHO)Food \,B \,+ \,\dots \dots \}] / \,Total \,g \,available \,CHO$$

The glycaemic load (GL) was calculated by multiplying the glycaemic index for each dietary product with carbohydrates in grams and dividing by 100, as shown in Eq. 3.

3 $$Glycaemic \,Load (GL) = Glycaemic \,Index (GI) X\,Grams \,of \,Carbohydrates \,(CHOg) \,/ 100$$

Pearson’s chi-square test, Fisher’s exact test, an independent T-test, and binary logistic regression models were applied to the variables, revealing significant differences between the cases and controls. Binary logistic regression analysis was applied to estimate the non-occurrence of PCOS as the predicted probability for physical activity level, glycaemic index, and glycaemic load.

### Ethical considerations

Ethical approval was obtained from the Punjab University Institutional Review Board (IRB # D/2022/UZ) and the Research and Advanced Studies Board (ASRB D- 5148/ACAD). Institutional Review Board approval was also obtained from the private hospital (IRB:104,010,295), and the participants provided informed consent before participating in the study, according to the Helsinki Declaration 2014 [27].

## Results

A total of 115 women with a mean age of 23.83 years (SD ± 5.19), a mean body mass index (BMI) of 23.35 kg/m^2^ (SD ± 4.58) and a mean waist-to-hip ratio (W/H) of 0.83 (SD ± 0.14) participated in this case–control study. The 49 newly identified PCOS cases had an average age of 24.63 years (SD ± 4.76), BMI 25.59 kg/m^2^ (SD ± 4.51), and W/H ratio of 0.85 (SD ± 0.17). The 66 healthy controls had an average age of 23.24 years (SD ± 5.45), BMI of 21.69 kg/m^2^ (SD ± 3.89), and W/H ratio of 0.83 (SD ± 0.11). The cases and controls were found to match (*p* > 0.05) for age, W/H, and year of menarche, but had a significant difference in BMI (*p* < 0.001). The average menarche age was 13.0 years (SD ± 1.45), with 61 (93.9%) of the controls having normal menstruation and 28 (57.1%) cases reporting oligo-ovulation. Polycystic ovarian morphology was evident among 36 (73.5%) of the cases, and a majority of them had a family history of PCOS (*n* = 25; 51.0%), hirsutism (*n* = 45; 91.8%), mFG score ≥ 10 (*n* = 42; 85.7%), and acne (*n* = 29; 59.2%) when evaluated using the Rotterdam criteria. Fisher’s exact test results showed that the reproductive characteristics of the cases were significantly different (*p* < 0.001) from those of the control group (Table 1).

**Table 1:** Demographic and reproductive characteristics of study participants

	**Group**	**Cases (*n* = 49)**	**Controls (*n* = 66)**	**Total (*n* = 115)**	**Fisher’s exact test**
					*p*-value
Age of participants Mean ± SD 23.83 ± 5.191	17–24 years	25 (51.0%)	43 (65.2%)	68 (59.1%)	0.134
	25–32 years	22 (44.9%)	18 (27.3%)	40 (34.8%)	
	33–40 years	2 (4.1%)	5 (7.6%)	7 (6.1%)	
BMI categories Mean ± SD 23.35 ± 4.58	Underweight	0 (0%)	16 (24.2%)	16 (13.9%)	< 0.001*
	Healthy weight	25 (51.0%)	36 (54.5%)	61 (53.0%)	
	Overweight	18 (36.7%)	13 (19.7%)	31 (27.0%)	
	Obese	6 (12.2%)	1 (1.5%)	7 (6.1%)	
Waist-to-Hip ratio Mean ± SD 0.83 ± 0.14	0.80 or below	15 (30.6%)	23 (34.8%)	38 (33.0%)	0.852
	0.81 to 0.85	13 (26.5%)	15 (22.7%)	28 (24.3%)	
	Greater than 0.85	21 (42.9%)	28 (42.4%)	49 (42.6%)	
Year of menarche Mean ± SD 13.0 ± 1.45	9–11 years	2 (4.1%)	11 (16.7%)	13 (11.3%)	0.072
	12–14 years	39 (79.6%)	49 (74.2%)	88 (76.5%)	
	15–16 years	8 (16.3%)	6 (9.1%)	14 (12.2%)	
Menstruation	Normal	11 (22.4%)	62 (93.9%)	73 (63.5%)	< 0.001*
	Anovulation	10 (20.4%)	3 (4.5%)	13 (11.3%)	
	Oligo-ovulation	28 (57.1%)	1 (1.5%)	29 (25.2%)	
Cyst	Yes	36 (73.5%)	0 (0%)	36 (31.3%)	< 0.001*
	No	13 (26.5%)	66 (100%)	79 (68.7%)	
Family history	Yes	25 (51.0%)	4 (6.1%)	29 (25.2%)	< 0.001*
	No	24 (49.0%)	62 (93.9%)	86 (74.8%)	
Hirsutism	Yes	45 (91.8%)	14 (21.2%)	59 (51.3%)	< 0.001*
	No	4 (8.2%)	52 (78.8%)	56 (48.7%)	
Acne	Yes	29 (59.2%)	16 (24.2%)	45 (39.1%)	< 0.001*
	No	20 (40.8%)	50 (75.8%)	70 (60.9%)	
mFG score	Less than 10	7 (14.3%)	48 (72.7%)	55 (47.8%)	< 0.001*
	Between 11–20	31 (63.3%)	17 (25.8%)	48 (41.7%)	
	Greater than 20	11 (22.4%)	1 (1.5%)	12 (10.45)	

^*^Significance level: *p*-value ≤ 0.05

The PCOS cases and healthy controls were compared for their daily physical activity and reported having moderate (30.6% vs. 56.1%) to high (38.8% vs. 39.4%) physical activity levels over the previous seven days. The cases and controls showed a significant difference (*p* < 0.001) in recorded physical activity levels. The energy consumption levels showed an average low energy consumption level of 780.8 METS-min per week (SD ± 710.2), moderate 2052.2 METS-min per week (SD ± 2014), and a vigorous 3840 METS-min per week (SD ± 382.4) for the selected participants. The mean comparison using an independent T-test for daily energy consumption levels showed a significant difference (*p* = 0.03) for low energy consumption levels between the two groups, as shown in Table 2. Binary logistic regression analysis showed that the odds of PCOS occurrence can be decreased by an increase in physical activity. A moderate physical activity level can prevent PCOS occurrence (OR = 6.84, 95% CI: 1.73–27.02; *p* = 0.006), and high physical activity can enhance the prevention of PCOS occurrence (OR = 12.33, 95% CI: 3.11–48.88; *p* < 0.001), as shown in Table 3.

**Table 2:** Comparison of physical activity and energy consumption levels between cases and controls

	**Group**	**Cases**	**Controls**	**Total**	***p*-value**
Weekly physical activity	Low (Walking)	15 (30.6%)	3 (4.5%)	18 (15.7%)	< 0.001*
	Moderate	15 (30.6%)	37 (56.1%)	52 (45.2%)	
	High	19 (38.8%)	26 (39.4%)	45 (39.1%)	
Low energy consumption level (MET) 780.8 ± 710.2 METS-min/week	Less than 1500 METS	47 (95.9%)	56 (84.8%)	103 (89.9%)	0.03*
	1501–3000 METS	2 (4.1%)	9 (13.6%)	11 (9.6%)	
	Greater than 3000 METS	0 (0%)	1 (1.5%)	1 (0.9%)	
Moderate energy consumption level (MET) 2052.2 ± 2014 METS-min/week	Less than 1500 METS	35 (30.4%)	36 (54.5%)	71 (61.7%)	0.25
	1501–3000 METS	5 (10.2%)	12 (18.2%)	17 (14.8%)	
	Greater than 3000 METS	9 (18.4%)	18 (24.8%)	27 (23.5%)	
Vigorous energy consumption level (METS) 3840 ± 382.4 METS-min/week	Less than 1500 METS	41 (83.7%)	61 (92.4%)	102 (88.7%)	0.40
	1501–3000 METS	7 (14.3%)	5 (7.6%)	12 (10.4%)	
	Greater than 3000 METS	1 (2.0%)	0 (0%)	1 (0.9%)	

^*^Significance level: *p*-value ≤ 0.05

**Table 3:** Binary logistic regression comparing the odds ratio for PCOS non-occurrence and physical activity levels

	**B**	**SE**	**Wald**	**df**	***p*-value**	**OR**	**95% CI for OR**	
							**Lower**	**Upper**
Physical activity levels			12.860	2	0.002*			
Moderate physical activity	1.923	0.701	7.531	1	0.006*	6.842	1.733	27.021
High physical activity	2.512	0.703	12.785	1	< 0.001*	12.333	3.112	48.884
Constant	-1.609	0.632	6.476	1	0.011	0.200		

^*^Significance level: *p*-value ≤ 0.05

A dietary intake comparison for macronutrients, vitamins, and minerals was made between the PCOS cases and healthy controls. The mean difference comparison showed that the cases and controls had significant differences for polyunsaturated fats (*p* = 0.05), cholesterol (*p* = 0.03), vitamin A, vitamin B6, vitamin B12, vitamin D, vitamin E, vitamin K, niacin, and thiamine (*p* ≤ 0.05). The glycaemic index (41.06 ± 4.00 vs. 40.58 ± 5.47) and glycaemic load (15.23 ± 6.52 vs. 17.27 ± 14.39) were found to be similar for the selected cases and controls (Table 4). A binary logistic regression analysis showed that food with a low glycaemic index (GI ≤ 40) reduced the odds of PCOS (OR = 1.94, 95% CI: 0.91–4.14; *p* = 0.085). Similarly, food nutrients with a low glycaemic load (GL ≤ 10) can reduce PCOS occurrence (OR = 1.60, 96% CI: 0.51–4.93; *p* = 0.414), as shown in Table 5.

**Table 4:** Mean difference comparison between dietary intake, glycaemic index, and glycaemic load

	**Variable**	**Cases**		**Controls**		***p*-value**
		**Mean**	**Standard Deviation**	**Mean**	**Standard Deviation**	
**Macronutrients**	Carbohydrates (g)	371.95	88.52	348.81	125.49	0.27
	Protein (g)	99.81	32.43	89.65	32.70	0.10
	Total Fat (g)	113.20	35.04	104.68	47.17	0.29
	Saturated fat (g)	39.49	13.88	34.66	15.38	0.09
	Monosaturated fat (g)	33.52	10.60	34.15	16.32	0.82
	Polyunsaturated fat (g)	37.19	11.06	31.94	15.96	0.05*
	Cholesterol (g)	246.60	98.66	207.94	88.74	0.03*
	Dietary Fiber (g)	20.92	4.43	19.30	7.62	0.18
**Vitamins**	Vitamin A (RE)	1804.90	725.16	1495.79	871.69	0.05*
	Vitamin B6 (mg)	1.79	0.47	1.50	0.56	< 0.01*
	Vitamin B12 (mcg)	5.29	2.24	4.08	2.11	< 0.01*
	Vitamin C (mg)	85.38	63.52	68.73	46.59	0.11
	Vitamin D (mcg)	161.11	91.50	121.10	99.35	0.03*
	Vitamin E (mg)	5.73	4.21	9.13	7.64	0.01*
	Vitamin K (mcg)	3.10	0.00	3.10	0.00	0.01*
	Niacin (mcg)	21.91	6.56	19.41	6.36	0.04*
	Riboflavin (mcg)	2.41	0.66	2.33	0.89	0.58
	Thiamin (mcg)	7.54	0.16	7.43	0.21	< 0.01*
	Folate	473.06	149.09	449.29	151.85	0.40
**Minerals**	Calcium (mg)	1242.63	462.45	1164.89	508.06	0.40
	Iron (mg)	35.47	8.20	32.24	10.50	0.08
	Magnesium (mg)	369.95	91.19	349.11	139.95	0.37
	Phosphate (mg)	1492.56	31.36	1478.13	42.57	0.05*
	Potassium (mg)	3274.26	809.42	2916.10	1034.80	0.05*
	Sodium (mg)	3791.74	1420.00	3535.89	1629.35	0.38
	Zinc (mg)	13.46	4.74	12.18	4.55	0.14
	Copper (mg)	1.58	0.45	1.57	0.60	0.97
	Magnese (mg)	43.91	22.36	33.96	24.63	0.03*
	Glycaemic index	41.06	4.00	40.58	5.47	0.60
	Glycaemic load	15.23	6.52	17.27	14.39	0.36

^*^Significance level: *p*-value ≤ 0.05

**Table 5:** Binary logistic regression to measure the odds ratio for PCOS occurrence glycaemic index

		B	SE	Wald	df	*p*-value	OR	95% CI for OR	
								Lower	Upper
Glycaemic Index Step 1^a^	Glycaemic Index less than 40	0.665	0.386	2.975	1	0.085	1.944	0.913	4.140
	Constant	0.000	0.254	0.000	1	1.000	1.000		
Glycaemic Load Step 1^b^	Glycaemic load categories			6.037	2	0.049			
	Glycaemic load less than 10	0.470	0.575	0.668	1	0.414	1.600	0.518	4.938
	Glycaemic load 10–20	-0.649	0.497	1.705	1	0.192	0.523	0.197	1.384
	Constant	0.511	0.422	1.468	1	0.226	1.667		

^a^Variable(s) entered on step 1: Glycaemic index greater than 40

^b^Variable(s) entered on step 1: Glycaemic load greater than 20 was considered as a reference value

## Discussion

The findings of this study show that physical activity levels and dietary patterns were different between women with PCOS and age-matched healthy controls. A significant difference was found between cases and controls in performing daily low levels of activity and a dietary intake with vitamin insufficiency. Similarly, in another nested case–control study, women with PCOS were found to be less physically active (*p* < 0.05) in their daily lives than healthy controls [28]. Women with PCOS were found to be less involved in sporting activities and reported to have a sedentary lifestyle, which can further lead to the development of metabolic syndrome [29]. A sedentary lifestyle and obesity among women with PCOS can be considered potential contributing factors to PCOM and hormonal abnormalities [30].

The study results show that increasing physical activity can gradually reduce PCOS occurrence, with moderate activity levels resulting in 6.8 times more preventive effect. A further increase in the intensity of daily physical activity can enhance these preventive effects 12-fold. Previous studies have also suggested that PCOS phenotype characteristics can be appraised by increasing daily physical activity levels as an independent therapy [31]. Multiple bouts of moderate physical activity level for at least 30 min each can help to achieve beneficial health effects among women with PCOS [32]. The gradual increase in physical activity can help to reduce inflammatory markers and can also reduce the risk of metabolic syndrome occurrence among women with PCOS [33]. Several studies have suggested that reproductive functions and metabolic characteristics improve with an increase in physical activity and weight management programmes among women with PCOS [34–36].

Dietary habits varied between selected PCOS cases and healthy women, particularly in terms of vitamin intake. A significant difference (*p* ≤ 0.05) was found for vitamin A, vitamin B6, vitamin B12, vitamin D, vitamin E, vitamin K, niacin, and thiamine. Women with PCOS consumed foods high in polyunsaturated fats and cholesterol. Similar evidence was reported in previous studies that women with PCOS had a dietary intake with higher polyunsaturated fat content and with a high energy load, which can further lead to dyslipidaemia and metabolic syndrome [37]. Dyslipidaemia, metabolic syndrome, insulin resistance, and diabetes mellitus type II have also been found to be common among women with PCOS [38–40]. It should be noted that the high amount of consumed vitamins, polyunsaturated fats, and cholesterol can result from a high intake of total energy.

Consumption of carbohydrates can be one of the important determinants of obesity and metabolic syndrome among women with PCOS. The role of carbohydrate consumption and its affinity with PCOS occurrence have attracted the attention of researchers. Women with PCOS consume high-calorie meals compared to healthy controls, but meals with a high glycaemic load have a negative impact on carbohydrate quality, despite no significant difference in macronutrient intake being observed in the present study. Research on the effectiveness of low-carbohydrate diets in managing PCOS remains inconclusive, despite some studies suggesting that such a diet may improve insulin sensitivity and regulate hormonal imbalances [41]. According to research, women with PCOS may experience carbohydrate cravings, which can lead to weight gain and metabolic syndrome, and greater GI intake is connected to obesity at a young age [42]. Further research is needed to understand the role of carbohydrates in PCOS development and symptoms, as individual variations and the multifaceted nature of the condition make definitive conclusions challenging.

Glycaemic index and glycaemic load are widely used parameters to evaluate the quantity and quality of carbohydrate content in the daily diet. Studies have shown that food with high GI and GL can cause hyperinsulinemia and a decrease in sex-hormone-binding globulin, which can cause ovarian dysfunction among women [43]. This study’s results found no significant difference between the cases and controls in carbohydrate consumption. However, the binary logistic regression model results showed that the probability of PCOS occurrence can be reduced with the consumption of food low in glycaemic index and glycaemic load.

The current study is novel in highlighting the public health issue of PCOS among women of reproductive age in Lahore, Pakistan. Profiling the physical activity levels and dietary patterns of women with PCOS and age-matched healthy controls gave a better understanding to compare the two groups. The newly diagnosed PCOS cases were identified using the Rotterdam criteria, which allowed the researchers to thoroughly examine these PCOS cases and compare them with healthy controls. The use of IPAQ and Mosby’s Nutritac v4.0 software enabled the complete and accurate evaluation of the physical activity levels and dietary habits of the participants. The findings from this study have established a possible link between individual lifestyle and PCOS occurrence, and future research is encouraged to conduct clinical trials to establish a causal relationship.

This study has the limitations of a small sample size and could only have an approximately 1:1 ratio of PCOS cases to healthy controls. A larger sample size with a 1:4 ratio between PCOS cases and healthy controls could yield further concrete findings. The researcher found it difficult to recruit healthy controls matched for both age and BMI in this study from the same healthcare centre.

## Conclusion

The individual’s lifestyle is a key determinant in the development and prognosis of PCOS. The daily physical activity levels and dietary habits of women of reproductive age can influence their reproductive characteristics and polycystic ovarian morphology. Women with PCOS had a high intake of macronutrients and low physical activity, which can contribute further to the development of obesity and related anomalies. Weight management programmes for PCOS should include low carbohydrate intake and the inclusion of moderate to high physical activity to obtain beneficial health effects on PCOS development.

### Supplementary Information

**Additional file 1.**

## Data Availability

The dataset used during the current study is available from the corresponding author on reasonable request.

## Abbreviations

ASRB: Research and Advanced Studies Board
BMI: Body Mass Index
CHO: Carbohydrates
FFQ: Food Frequency Questionnaire
GI: Glycaemic index
GL: Glycaemic load
IPAQ: Physical Activity Questionnaire
IR: Insulin Resistance
IRB: Institutional Review Board
LHW: Lady Health Worker
M: Mean
METs: Metabolic Rate
mFG: Modified Ferriman-Gallwey
O.R: Odds Ratio
PCOM: Polycystic Ovaries Morphology
PCOS: Polycystic Ovarian Syndrome
SD: Standard Deviations
TVS: Transvaginal Sonogram
VO2 max: Maximum Oxygen Consumption
W/H: Waist-To-Hip Ratio
WC: Waist Circumference
