# Supplementary material for: Comparison of physical activity levels and dietary habits between women with polycystic ovarian syndrome and healthy controls of reproductive age: a case-control study
Source: BMC Womens Health. 2024 Jan 8;24:29. doi: 10.1186/s12905-023-02866-3 (PMC10775673; doi:10.1186/s12905-023-02866-3)
Supplement: Supplementary file 1 — Additional file 1. [file 12905_2023_2866_MOESM1_ESM.pdf]

Supplementary material

7-Days Food Frequency Questionnaire with estimated reference values in grams for one serving and relevant food labels in Mosby's Nutritrac nutrition									
Food Category	Food Groups	Food Label in Mosby's Nutritrac nutrition analysis	Estimated amount in grams for one serving	How often did you eat in the last 7 days				For Calculations	
				Do you eat this food	No of days in a week when this food item is eaten	On the days when you eat this food how many times in a day you eat it	No of servings eaten at one occasion	Number of one serving calculated	Weight of one serving calculated
				Y/N					
MEAT	Beef	Gravy Beef	100 g						
	Mutton/Lamb	Lamb, Meat and Fat Cooked	100 g						
	Chicken	Gravy, Chicken, Cooked	100 g						
	Oil or Ghee	Oil, vegetables, canola	100 ml						
FISH AND POULTRY	Fish	Fast Food Fish	100 g						
	Prawns/Shrimps	Prawn	100 g						
	Eggs	Egg and Bread	100 g						
PULSES	Bean	Bean	193 g						
	Lentils	Lentils	198 g						
CEREALS	Rice (Cooked)	Rice, white, Long-grain	158 g						
	Pasta/Noodles	Pasta, Homemade, with Egg	128 g						
	Bread	Bread, Mixed-grain	28.4 g						
	Potato	Potato, Homemade	75 g						
DAIRY PRODUCTS	Whole Milk	Milk	244 ml						
	Milk without cream	Milk substitute	244 ml						
	Milk dessert	Pudding, Vanilla	313 ml						
	Cream	Cream and Milk	3 ml						

	Yogurt	Frozen Yogurt	170 ml						
	Lassi	Yogurt, Plain, Whole Milk	170 ml						
	Salty lassi	Yogurt, Skim Milk	170 ml						
FATS	Margarine	Margarine, Regular w/salt	14.2 g						
	Butter	Butter with Salt	14.2 g						
SNACKS	Fried Snacks	Chips, Potato,	117 g						
	Processed Food items	Cheese Spread, Processed	78 g						
VEGETABLES	Raw vegetables	Vegetables, Mixed	91 g						
	Cooked vegetables	Vegetables, Mixed	91 g						
FRUITS	Fresh Fruits	Fruit Salad, Juice Pack	249 g						
SWEETS	Chocolate	Cookies, Wafers, Chocolate	24 g						
	Pakistani desserts	Raisins, Chocolate, Milk,	30 g						
	Bakery products	Biscuits, Plain or buttermilk	30 g						
BEVERAGES	Fresh fruit juices	Juice, Tropical Fruits	250 ml						
	Soft drinks	Coca Cola	250 ml						
NUTS	Seasonal Nuts	Almonds, Dry Roasted	29 ml						
